# Seasonal Variations in Heart Rate Variability as an Indicator of Stress in Free-Ranging Pregnant Przewalski's Horses (*E. ferus przewalskii*) within the Hortobágy National Park in Hungary

**DOI:** 10.3389/fphys.2017.00664

**Published:** 2017-09-07

**Authors:** Friederike Pohlin, Kristin Brabender, Gerhard Fluch, Gabrielle Stalder, Thierry Petit, Chris Walzer

**Affiliations:** ^1^Department of Integrative Biology and Evolution, Research Institute of Wildlife Ecology, University of Veterinary Medicine Vienna Vienna, Austria; ^2^Hortobagy National Park Hortobágy, Hungary; ^3^Zoo de la Palmyre Les Mathes, France; ^4^Wildlife Conservation Society Bronx, NY, United States

**Keywords:** adaptation, allostatic load, *Equus ferus przewalskii*, heart rate variability, parasympathetic nervous system, Przewalski's horse, seasonal variations, stress

## Abstract

**Background:** Ecosystems with seasonal fluctuations in climate and food availability present physiological challenges to resident mammals and may cause “stress.” The two predominant physiological responses to stressors are (1) the activation of the hypothalamic-pituitary-adrenal axis and (2) the modulation of the autonomic nervous system. To date, the primary indicator for “stress” in wildlife- and zoo animal research are glucocorticoid levels. By measuring the autonomic regulation of cardiac activity, particularly the vagal tone, heart rate variability (HRV) is presently emerging as a suitable indicator of “stress” in farm- and domestic animal research.

**Objective:** The aim of this study was to use HRV, a novel method in wildlife research, to assess seasonal patterns of “stress” in a group of free-ranging Przewalski's horses (*Equus ferus przewalskii*).

**Methods:** Six pregnant Przewalski's horses from one harem within the Hortobágy National Park in Hungary were subjected to the study. We used a dedicated telemetry system consisting of a subcutaneously implanted transmitter and a receiver and storage unit in a collar to record HRV, heart rate (HR), subcutaneous body temperature, and activity throughout a one-year study period—climate data was also collected. We defined “stress” as a decrease in parasympathetic nervous system tone and calculated RMSSD (root mean square of successive differences) as a measure of HRV. Linear mixed effects models with random intercept per individual were used for statistical analysis.

**Results:** HRV and HR varied considerably throughout the year. Similar to temperate ruminants and hibernating mammals, Przewalski's horses experienced lower HR and HRV during winter, when resources are limited indicating decreased metabolic rates coupled with “stress.” In spring, we observed a drop of HRV along with a peak in HR indicating an increase of allostatic load that is most likely associated with increased energy demands during pregnancy and/or seasonal routines such as the adjustment of the gastrointestinal system to better quality diet.

**Conclusion:** Measuring telemetric HRV is a proven method to study undisturbed reactions of wild animals to their changing environment over the long term. Przewalski's horses experience a loss of complexity in cardiovascular dynamics over the winter and particularly during spring, indicating seasonal “stress.”

## Introduction

In seasonal environments, free-ranging organisms experience regular patterns of physiological and behavioral responses that allow the individual to cope with predictable environmental changes (Romero, 2002; McEwen and Wingfield, 2003; Boonstra, 2004; Reeder and Kramer, 2005). Measuring “stress” allows us to quantify these responses and to investigate how organisms integrate their life cycles in the natural world—knowledge critical for conservation physiology (Wikelski and Cooke, 2006; Tarlow and Blumstein, 2007; Busch and Hayward, 2009; Dantzer et al., 2014).

“Stress” was first described by Hans Selye in 1936, who defined it as a non-specific response of the body to any demand for change (Selye, 1936). Later, the concept has been refined by introducing the terms “stressor” and “stress response.” A stressor is a stimulus that threatens homeostasis (stability of physiological variables), whereas the stress response is the reaction of the organism to regain homeostasis (Chrousos, 2009). “Stress” would then be a state in which homeostasis is threatened. The hypothalamic-pituitary-adrenocortical (HPA) axis and the autonomic nervous system are the two key-players of the stress response (McEwen and Wingfield, 2003; Reeder and Kramer, 2005; Koolhaas et al., 2011). These systems respond to unpredictable stressful events in daily life, but also to predictable challenges throughout the day and year.

To date, measuring “stress” in wildlife has mainly focused on the quantification of glucocorticoid steroid hormones (i.e., cortisol and corticosterone), the primary mediators of the HPA axis (Möstl and Palme, 2002; Millspaugh and Washburn, 2004; Schwarzenberger, 2007; Busch and Hayward, 2009; Sheriff et al., 2011; Dantzer et al., 2014). In human medicine and farm- and companion animal research, heart rate variability (HRV) has been used increasingly over the past decades to evaluate physical and psychological stress (Mohr et al., 2002; Pumprla et al., 2002; VonBorell et al., 2007; Stucke et al., 2015). HRV quantifies the constantly changing time intervals between consecutive heartbeats (NN-intervals). This means that it is based on the antagonistic oscillatory influences of the parasympathetic- and sympathetic-nervous system on the heart and thus reflects the functioning of the autonomic nervous system (Malik et al., 1996; Pumprla et al., 2002; VonBorell et al., 2007).

Whilst the parasympathetic nervous system promotes homeostasis and optimizes the function of internal viscera, the sympathetic nervous system responds to challenges from outside the body. In an attempt to redefine “stress,” Porges (1995a) proposed a model based on the tradeoff between internal and external needs—*when internal needs* (homeostasis) *are no longer being adequately serviced by the parasympathetic nervous system, the organism is experiencing stress* (Porges, 1995a). Thus, “stress” can be investigated by measuring parasympathetic nervous system function.

Compared to the sympathetic nervous system, the parasympathetic modulation of the heart is much faster (Pumprla et al., 2002). Various HRV measures that reflect these rapid changes, and hence cardiac vagal activity, have recently been reviewed in order to determine the best method to characterize parasympathetic nervous system function (Laborde et al., 2017). Of all measures, root mean square of successive differences (RMSSD) has been considered the preferred index under free-running conditions, as it is relatively free from respiratory influences (Penttila et al., 2001; Hill and Siebenbrock, 2009; Saboul et al., 2013) and fairly easy to calculate and interpret (Malik et al., 1996; VonBorell et al., 2007; Plews et al., 2013).

In this study, we investigated seasonal patterns of “stress” in a group of free-ranging Przewalski's horses (*Equus ferus przewalskii*) within the Hortobágy National Park in Hungary by measuring HRV—a novel method in wildlife research. We employed RMSSD as an indicator for parasympathetic nervous system tone and measure of “stress.”

The Przewalski's horse (*Equus ferus przewalskii* Poljakov, 1881) is a typical steppe herbivore whose distribution once covered the entire Eurasian steppe belt (Wakefield et al., 2002). Pasture competition with livestock and over-hunting led to the horses moving east to Asia, and eventually becoming extinct in the wild in the 1960s (Boyd and Houpt, 1994; Wakefield et al., 2002). The species has been saved from total extinction by breeding in captivity (Ryder and Wedemeyer, 1982; Ryder, 1993) and reintroduction into its formal range in Mongolia (Wakefield et al., 2002; King et al., 2015). There, temperatures range from −40° to +40°C and water resources are limited (Boyd and Houpt, 1994). Semi reserves, such as the Hortobágy National Park in Hungary, were created to prepare horses for release into the wild and to offer opportunities for research (Zimmermann, 2005)—understanding the species' physiological capabilities and seasonal adjustments is critical for the successful management of reintroduced animals and hence, conservation of the Przewalski's horse.

In order to observe undisturbed reactions of free-ranging Przewalski's horses to their changing environment over the long term, we recorded cardiac activity with a transmitter-implant under the skin of the horses. We hypothesized that HRV would be highest during the summer and lowest during the winter, indicating seasonal “stress” due to energetic bottlenecks.

## Materials and methods

### Study site

The study was performed in the Pentezug region of the semi reserve Hortobágy National Park in Hungary. Pentezug is a steppe region of 2,388 ha (27 ha forest, 2,361 ha meadow) that is under the highest level of protection. It is closed to the public and any agricultural activity that might cause damage to the sensitive fauna and flora is prohibited. At present, ~300 Przewalski's horses freely roam the grasslands of the park representing the largest population in the world. The horses live in harem units, consisting of one mature breeding stallion and up to eight adult mares with their offspring. Female horses frequently give birth to one foal per year and, as there are no predators in the park, the population is increasing. In fact, more than 50 foals are currently being born annually (Zimmermann et al., 2009b; Makra, 2016). Average temperature within the park ranges around 21°C in the summer and −2.5°C in the winter. The average annual rainfall is ~500 mm, and snowfall ~2–10 cm, distributed throughout 40–45 days per year (Zimmermann et al., 2009a). The horses depend entirely on the natural vegetation and are fully exposed to the changing climatic conditions.

### Data collection

The study and all procedures were performed 2008 in Hungary. The responsible veterinary administration authorities in the Hajdú-Bihar County approved the captures and subsequent procedures according to the Act on Animal Protection 1998. In 2008, there was no provision for the Animal Welfare and Ethics Committee of the Vetmeduni Vienna to review or approve projects carried out outside of Austria.

In October 2008, we captured six adult Przewalski's horse mares (aged 3–11 years old; mean ± *SD* age, 6 ± 3 years; mean estimated weight, 295 ± *SD* 15 kg; range, 270–320 kg) by remote darting from foot or vehicle. Only female horses from one harem were selected for two reasons: (1) their behavior is synchronized reducing interindividual variability (Souris et al., 2007), (2) stallions tend to break valuable monitoring equipment due to their aggressive behavior (Kolter and Zimmermann, 2001). All of the six mares were pregnant and gave birth to healthy foals during the study period (6th May–7th June 2009; mean 23rd May ± *SD* 5 days).

A combination of 10 mg butorphanol (Torbugesic, Fort Dodge Animal Health, Fort Dodge, Iowa 50501, USA), 10 mg detomidine (Domosedan, Orion Corp. Farmos Finland), and 0.7–1.4 mg ethorphine (M99, C-Vet Veterinary Products, Lancs, UK) was used to induce anesthesia. Anesthesia was maintained with an intravenous infusion of guaifenesin-ketamine-xylazine [1 L of 5% guaifenesin (Myolaxin, Vétoquinol UK Ltd, Buckingham, MK18 1PA, UK) 1,000 mg ketamine (Ketamidor, Richter Pharma, 4600 Wels, Austria) and 500 mg xylazine (Rompun, Bayer Austria Ges.m.b.H, 1160 Vienna, Austria)]. For further details regarding anesthesia, see Walzer et al. (2009).

**Figure 1:**
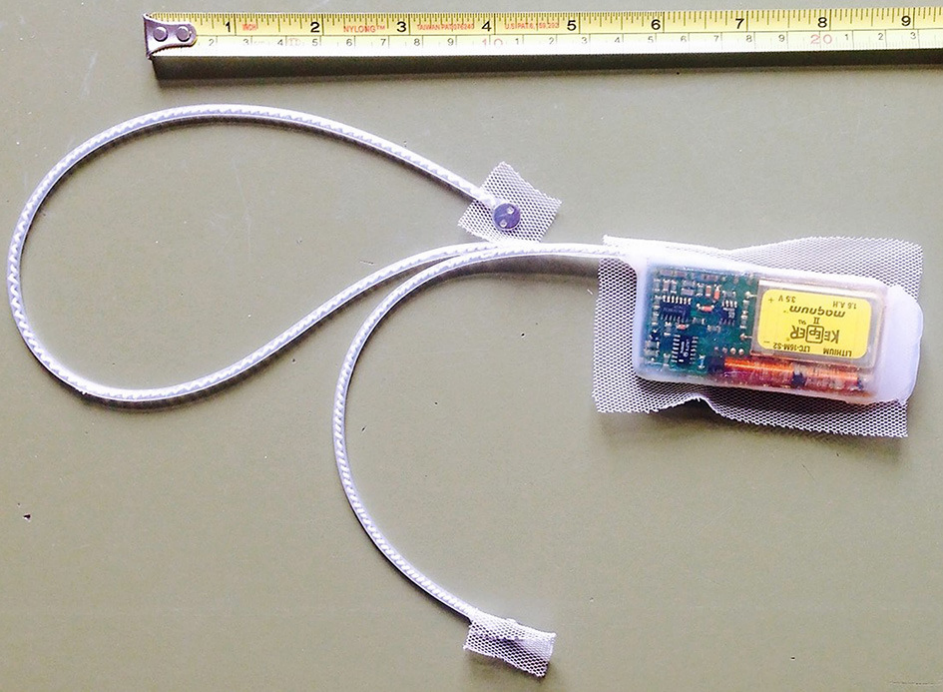
Photograph showing the self-constructed transmitter with the two electrode plates. We implanted the transmitter under the skin of the left side of the neck. Electrodes were placed next to the sternum.

The horses were fitted with collars, which included a dual-axis motion sensor to monitor activity, and very high frequency (VHF) transmitters for tracking purposes. In order to measure cardiac activity, we used a system consisting of an implantable transmitter unit with two electrodes (Figure 1), and a receiver and storage unit located in the collar. The microprocessor-controlled transmitter (50 g, 65 × 35 × 11 mm) was encapsulated in physiologically inert medical-grade silicone rubber and housed a thermistor. Two electrode plates (surgical steel, 8 mm diameter) were connected to the transmitter with a coiled silicone rubber-insulated wire of multi-stranded stainless steel fitted in silicone rubber tubing. We surgically implanted the transmitter between the muscle and subcutaneous fat on the ventro-lateral aspect on the left side of the neck. Two small caudo-ventral incisions served to pull the electrodes subcutaneously from the transmitter to a peristernal position. Once instrumentation was in place, we affixed the transmitter and the electrodes to the subcutaneous tissue and closed the skin incisions with absorbable suture material. The implantable unit recorded heart rate (HR) and subcutaneous body temperature at intervals of 1 min. Every 15 min, cardiac activity was recorded on a beat-to-beat basis for a time window of 3 min. Resolution of body temperature was 0.01°C, and of cardiac measurements beats per minute (bpm).

The parameters were transmitted at 100 kHz via a short-range telemetry data link to the receiver and storage unit in the collar. We obtained hourly climate data (outdoor temperature, wind, rain, and humidity) from a weather station located in the study area.

Data was retrieved from the collars 18 months after instrumentation. One horse lost its collar 3 months after instrumentation (it was therefore excluded from the study) and two horses at ~10 months.

### Data analysis

The raw, telemetrically obtained, data contained obviously erroneous values due to electronic noise and disturbance during transmission. We removed all HR-values outside the described physiological range for horses of 10–250 bpm from the database. We computed the corresponding NN-intervals and converted the values into milliseconds (i.e., divided 60 through the HR, and multiplied the resulting values with 1,000). In order to clean the data from transmission errors and other noise, we removed sessions with <30 measurements and removed NN-interval values that deviated more than two standard deviations from the respective session mean. To avoid a bias resulting from handling and surgery, the first 2 weeks after anesthesia and surgery were discarded from statistical analysis. As the duration of recordings varied between the individuals, data of no longer than 12 months were included.

We determined HRV by calculating the square root of the mean of the sum of the squares of differences between adjacent NN intervals (RMSSD). It is recommended to perform basal cardiac measurements in horses only under resting conditions, when parasympathetic activity is high (Parker et al., 2010; Stucke et al., 2015). Therefore, we first evaluated RMSSD throughout the 24-h period of the day and used only data from the 6-h period of the day with the highest values for further analysis.

Statistical tests were performed using R 3.3.1 for Windows (The R Foundation, Vienna, Austria). We used a linear mixed-effects model to assess the effect of two continuous independent climate variables (outdoor temperature and humidity), two binary independent climate variables (rain and wind), two continuous independent physiological variables (HR and body temperature), and one binary independent physiological variable (activity) on one continuous dependent variable (RMSSD) for all Przewalski's mares. The mixed-effects model allows repeated measures by including a random intercept for each subject (each horse). Standardized scores were used for continuous variables, and RMSSD was log-transformed to account for its heavily skewed distribution. We fit multiple nested models including (1) random intercepts for each horse and a fixed effect for HR and month. We then stepwise added fixed effects for (2) activity and body temperature, (3) climate data, and (4) a quadratic effect of outdoor temperature. Akaike's Information Criterion (AIC) was used to compare the goodness of fit for the set of models.

## Results

**Figure 2:**
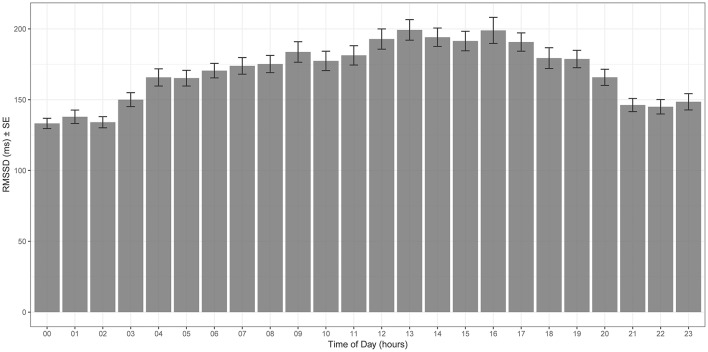
Hourly mean RMSSD throughout the 12 months study period [x-axis time of the day (hour), y-axis RMSSD (ms)]. RMSSD is highest during the third quarter of the day (12 p.m. to 6 p.m.).

Mean values of RMSSD for each hour of the day are plotted in Figure 2—RMSSD was highest between 12 p.m. and 6 p.m., the third quarter of the day, and lowest at around midnight.

**Figure 3:**
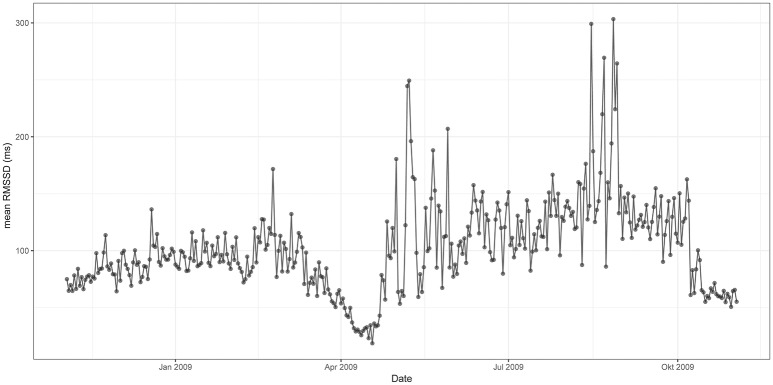
Mean RMSSD from 12 p.m. to 6 p.m. throughout the 12 months study period [x-axis date (month), y-axis RMSSD (ms)]. RMSSD is lower during the winter than during the summer—it experiences a drop in spring.

**Figure 4:**
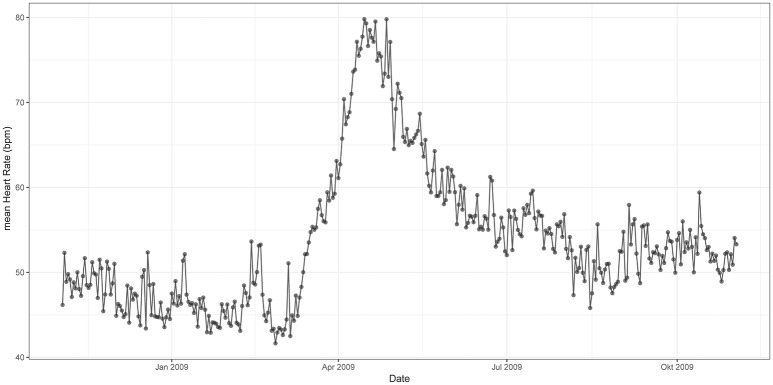
Mean HR from 12 p.m. to 6 p.m. throughout the 12 months study period [x-axis date (month), y-axis HR (bpm)]. HR is lower during the winter than during the summer—it experiences a peak in spring.

Figures 3, 4 represent mean values of RMSSD (Figure 3) and HR (Figure 4) for the third quarter of each day of the year (individual values are represented in Supplementary information: Figures 1, 2). Cardiac activity varied considerably throughout the year. RMSSD and HR showed lower values during the winter than during the summer. RMSSD dropped in April along with a concurrent peak in HR.

When compared to January, RMSSD experienced a pronounced drop in April. It increased rapidly thereafter and remained on significantly higher levels from June to October (mean 120 ms) whereby highest values were observed in August. RMSSD decreased in fall, and remained lower (mean 87 ms) during the winter-months when compared to the summer months (Figure 3).

In contrast, HR increased considerably during March and experienced a pronounced peak in April. This peak was concurrent with the observed drop in RMSSD. After a rapid decrease, HR remained on an intermediate level (mean 50 bpm) during summer and further declined during autumn to reach a mean of ~45 bpm during the winter. The total annual change was approximately twofold, with a maximum of 80 bpm in spring (Figure 4).

RMSSD correlated negatively with HR and body temperature, and positively with activity. Regarding climatic effects, RMSSD correlated positively with rain. Humidity showed a small positive effect on RMSSD, whereas wind had no significant effect. Outdoor temperature had a negative effect on RMSSD—this effect was quadratic: temperatures of around 18°C were associated with minimum RMSSD values. The model including the quadratic effect of outdoor temperature showed the lowest AIC (i.e., the best fit, see Supplementary Table 1). In summary, the energetic consequences of thermoregulation and weather conditions contributed significantly to the seasonal changes in RMSSD (Table 1). Of all physiological and climatic parameters, activity had the strongest effect on RMSSD. HR only accounted for ~2.5% of variation.

**Table 1:** Factors associated with seasonal variation of daily mean RMSSD.

	**RMSSD**			
	***Coefficient***	***Confidence Interval***	***Std. Error***	***p-value***
**FIXED EFFECTS**				
(Intercept)	−0.38	−0.90 to 0.15	0.27	0.157
Heart Rate	−0.16	−0.17 to −0.14	0.01	<0.001
Activity	0.35	0.32 to 0.38	0.02	<0.001
Body Temperature	−0.04	−0.06 to −0.03	0.01	<0.001
Rain	0.15	0.10 to 0.20	0.03	<0.001
Wind	0.01	−0.01 to 0.04	0.01	0.172
Outdoor Temperature	−0.12	−0.14 to −0.09	0.01	<0.001
Outdoor Temperature^2^	0.06	0.05 to 0.07	0.01	<0.001
Humidity	0.03	0.02 to 0.05	0.01	<0.001
Month (Feb)	0.14	0.10 to 0.19	0.02	<0.001
Month (Mar)	−0.03	−0.08 to 0.02	0.03	0.245
Month (Apr)	−0.75	−0.82 to −0.67	0.04	<0.001
Month (May)	0.01	−0.06 to 0.08	0.04	0.808
Month (Jun)	0.23	0.16 to 0.30	0.04	<0.001
Month (Jul)	0.30	0.22 to 0.38	0.04	<0.001
Month (Aug)	0.85	0.74 to 0.96	0.06	<0.001
Month (Sep)	0.34	0.27 to 0.42	0.04	<0.001
Month (Oct)	0.21	0.14 to 0.27	0.03	<0.001
Month (Nov)	−0.02	−0.07 to 0.03	0.03	0.439
Month (Dec)	0.12	0.07 to 0.17	0.03	<0.001
**RANDOM EFFECTS**				
σ^2^	0.577			
τ_00, Implant_	0.353			
N_Implant_	5			
ICC_Implant_	0.380			
Observations	26671			
R^2^/$\Omega_{0}^{2}$	0.522/0.522			

*The table provides the fixed and random effects for our final model. Activity, Rain, and Wind are coded as binary variables (i.e., “present” or “not present”). Heart Rate, Body Temperature, Humidity, and Outdoor Temperature are standardized and normally distributed. Our dependent variable is RMSSD, which we log-transformed to account for its highly skewed distribution*.

## Discussion

This is the first study using HRV to measure seasonal patterns of “stress” in free-ranging wildlife. During the past decades, measuring fecal glucocorticoid metabolite levels has become the method of choice to non-invasively index “stress” in zoo- and wild animal research (Möstl and Palme, 2002; Touma and Palme, 2005; Schwarzenberger, 2007; Sheriff et al., 2011; Dantzer et al., 2014; Hadinger et al., 2015). However, long-term recordings with short sampling intervals are often logistically difficult. Samples collected under field conditions cannot always be preserved immediately, the length of time between defecation and collection is often unknown, and assignment of the sample to an individual might be difficult, all factors influencing final results (Huber et al., 2003; Sheriff et al., 2011; Hadinger et al., 2015). HR is another parameter that has been used to study physiological reactions of free-ranging wildlife to environmental stressors (MacArthur et al., 1979; Harlow et al., 1987; Weisenberger et al., 1996; Ackerman et al., 2004; Theil et al., 2004; Arnold et al., 2006; Laske et al., 2011). With advancements in technology, transmitters that include cardiac monitors have become smaller and less invasive, while the monitoring period has increased from days to months to years (White and Garrott, 1990; Rutz and Hays, 2009). Although, HR measurement has been applied to assess short-term effects of “stress,” it more exactly represents an indicator for metabolic rate and energy expenditure (Weimerskirch et al., 2002; Butler et al., 2004; Groscolas et al., 2010; Green, 2011). By measuring the autonomic regulation of cardiac activity, particularly the vagal tone, HRV is now progressively emerging as a suitable indicator of “stress” in farm- and domestic animal research (VonBorell et al., 2007). In wildlife research, it is a fairly new approach, and only few previous studies have applied it (Theil et al., 2004; Mentaberre et al., 2010; Støen et al., 2015; Evans et al., 2016). All of these studies used SDANN (standard deviation of the average NN interval calculated over short periods, usually 5 min) as a measure of HRV. In contrast, we applied RMSSD (the square root of the mean squared differences of successive NN intervals) as a measure of HRV. Our aim was to assess seasonal variations in parasympathetic nervous system activity and hence, “stress” in free ranging Przewalski's horses. Whereas SDANN represents a general measurement of vagal and sympathetic influences on the heart, RMSSD specifically reflects vagally mediated alterations in the autonomic nervous system (Malik et al., 1996; VonBorell et al., 2007). SDANN is easier to calculate, but RMSSD has superior statistical properties (Malik et al., 1996). In our study, HRV was recorded in sequences of 3 min. The Task Force of The European Society of Cardiology and The North American Society of Pacing and Electrophysiology recommends recordings of at least 5-min-intervals to generate accurate HRV measurements (Malik et al., 1996). However, it has been shown that RMSSD, but not SDANN, can reliably be assessed from shorter recordings (Nussinovitch et al., 2011; Plews et al., 2013; Esco and Flatt, 2014; Munoz et al., 2015). Given our hypothesis and the experimental setting, RMSSD was the better parameter to choose. Parker et al. (2010) compared an automatic RR-detector (Polar®S810) with a gold standard ECG in horses and described failure to correctly identify one or more R-peaks. Different electrode types might explain the different signal quality between the two systems. In our study, electrodes were implanted under the skin of the horses, providing greater contact than conventional RR-detectors. To date, there are no studies comparing insertable cardiac monitors with conventional ECG in horses or other animals. The assessment of sensitivity of the current method was outside the scope of the study, but would be of great future interest.

In domestic horses, it is recommended to perform basal cardiac measurements only under resting conditions (Parker et al., 2010; Stucke et al., 2015). We overcame this problem by documenting diurnal variations in RMSSD. We identified the quarter of the day with the highest RMSSD values and included only data from that respective quarter of the day in statistical analysis.

We found that free-ranging Przewalski's horses exhibited diurnal variations of the autonomic nervous system function. RMSSD values were highest during the third quarter of the day (12 p.m.–6 p.m.), indicating an increased parasympathetic tone. At around midnight, parasympathetic tone was lowest. Accordingly, a peak in resting behavior in the middle of the day and a more vigilant and reactive behavior during the night has been observed in Przewalski's horses under natural conditions (Berger et al., 1999; Souris et al., 2007; King et al., 2016). Circadian patterns of HRV have been described in only a few domestic animal species. In contrast to our study, parasympathetic tone was higher during the night and lower during the day in most domestic species, possibly reflecting adaptation to human diurnality (Kuwahara et al., 1999a,b; Murphy, 2010; Gehrke et al., 2011; Kovács et al., 2016). Photoperiod is known to strongly influence daily and seasonal cycles in autonomic nervous system function (Buijs et al., 2003; Hastings et al., 2007; Vandewalle, 2007). However, the examination of the possible association between day-length and HRV was beyond the scope of this study.

RMSSD and HR varied considerably throughout the year. Both parameters showed lower values during the winter than during the summer. In spring, RMSSD dropped significantly along with a concurrent peak in HR.

The lower levels of RMSSD during winter and particularly the decrease during spring indicate a considerable reduction of the vagal tone. This is in agreement with the main assumptions of the polyvagal theory of Porges (1995a,b, 2007), who considered the vagal nerve as a mediator in reaction to stress. According to the theory, the vagal nerve is distinct with two different branches: (1) the myelinated vagus that is associated with attention, motion, and emotion, and (2) the unmyelinated vagus that mediates the reflexive regulation of visceral function. Both branches may have different outputs to the heart, but both branches can cause bradycardia. Upon reduction of the myelinated vagal tone, HR increases. At the same time, cardiac pacemakers are still prone to neurogenic bradycardia mediated by the unmyelinated vagus (Porges, 1995b, 2007). This means that, even though HR is lower during the winter, the organism can still experience “stress.”

HR correlates with cardiac output and oxygen consumption. Therefore, it can be used as an indicator for energy expenditure (Butler et al., 2004; Green, 2011). The Przewalski's horses in our study had a lower metabolic rate during the winter than during the summer. In spring, there was a peak in energy expenditure. A low metabolic rate during the winter and resumption of high metabolic activity in spring are well known from hibernators and species exhibiting daily torpor (Heldmaier et al., 2004; Evans et al., 2016). This phenomenon has also been previously described in a number of other non-hibernating mammal species (Weiner, 1977; Moen, 1978; Mesteig et al., 2000; Arnold et al., 2004; Theil et al., 2004), including the Przewalski's horse in the National Park Neusiedlersee-Seewinkel in Austria (Arnold et al., 2006). It is believed that these mammals, reduce body mass and organ size during the winter, and decrease endogenous heat production to be able to cope with predictable energetic bottlenecks (Ruf and Heldmaier, 1992; Arnold et al., 2004; Heldmaier et al., 2004).

In a recent study, Evans et al. (2016) investigated the drivers of hibernation in the Scandinavian brown bear. Amongst others, they examined HR and HRV (by measuring SDANN) throughout the year and found, similar to our study, that both parameters were significantly lower during the winter than during the summer. In our study, HRV correlated negatively with HR and body temperature. This is not surprising as an increase in HR results, in most cases, from a combination of reduced vagal activity (measured as a decrease in RMSSD) and increased sympathetic activity (VonBorell et al., 2007; Battipaglia and Lanza, 2015). The associated increase in metabolic heat production leads to an elevation of body temperature (Buller et al., 2013; Sim et al., 2014). In our (linear) model with log-transformed RMSSD, HR only accounted for ~2.5% of the observed variance. The primary reason to log-transform the raw RMSSD scores was to account for its skewed distribution, and this could have influenced the relationship between our HRV and HR measures. It would be interesting to explore this further with different operationalization of HRV in future research.

Of all physiologic parameters measured in our study, activity had the highest effect on HRV. When horses were active, RMSSD increased indicating that horses were more relaxed when moving as compared to standing still. Accordingly, Theil et al. (2004) described highest HRV values for locomotive behavior in European roe deer. In domestic horses in contrast, HRV increased from rest to walk, but decreased from walk to trot and gallop (Physick-Sheard et al., 2000; Kinnunen et al., 2006).

Theil et al. (2004) further described a positive effect of wind speed on HRV in European roe deer. Of all climatic parameters measured in this study, wind had no significant effect on RMSSD. Rain in contrast, was associated with a significant increase in RMSSD. Precipitation is the single most important climatic variable controlling the ecology of semiarid steppe-regions, the Przewalski's horse's habitat (Boyd and Houpt, 1994; Zimmermann et al., 2009a; Werger and VanStaalduinen, 2012). Hence, rainfall highly influences vegetation quality and water availability (Lauenroth and Sala, 1992; Kuntz et al., 2006), and might therefore indirectly affect HRV (Singh et al., 2000).

Outdoor temperature in contrast had a negative effect on HRV indicating that horses might be more vulnerable to heat-stress than to cold-stress. However, the effect of outdoor temperature was quadratic: minimum values of RMSSD were associated with temperatures ranging at around 18°C. This is the case during spring, when we observed a pronounced drop in HRV along with a peak in HR. A peak in metabolic rate during May has been described in European roe deer (Theil et al., 2004) and free-ranging Przewalski's horses within the National Park Neusiedlersee-Seewinkel in Austria (Arnold et al., 2006). Theil et al. (2004) suggested that this peak probably reflects the increased energetic costs of gestation and preparation for lactation. As all horses in our study were pregnant, reproduction might be a possible cause for the increased energy demands observed in April. Additional load on the cardiovascular system, hormonal control and induction of insulin resistance to provide energy to the growing fetus result in metabolic stress (Fowden et al., 1984; Pashen, 1984; Boyd and Houpt, 1994; Satué and Domingo, 2011). Interestingly, the horses of our study did not give birth until 1 month after the observed drop in HRV and peak in HR. Likewise, the roe deer in Theil et al. (2004) study only gave birth in early June whereas the peak took already place in May. This could be explained by a pronounced increase in fetal size during this stage of pregnancy in both species (Robbins and Robbins, 1979; Platt, 1984). However, domestic horses have been shown to only exhibit a slight increase in HR and no significant changes in RMSSD toward the end of pregnancy and postpartum (Nagel et al., 2011, 2012). Furthermore, Arnold et al. (2006) described a May peak in HR in a mare that only foaled in August. Thus, higher energy expenditure for gestation does not seem to entirely explain the drop of parasympathetic nervous system activity and peak of metabolic rate during spring. Kuntz et al. (2006) argued that seasonal changes in forage composition might be the driver of respective physiological changes in the Przewalski's horses. As crude protein increases during the vegetative period in spring, dry matter intake increases and hence, energy intake. After being in a katabolic state over the winter, horses have to adapt, amongst others, their gastrointestinal system to the higher quality forage and increase food intake to restore energy balance (Arnold et al., 2006; Kuntz et al., 2006). These metabolic changes have been found to occur prior to seasonal changes in plant phenology, suggesting an endogenous control mechanism preparing the organism in advance to the predictable seasonal changes in food quality (Arnold et al., 2006). In order to understand the role of pregnancy within these metabolic changes, seasonal variations in HR and HRV need to be studied in male and non-pregnant female horses.

For decades, demands associated with predictable seasonal variations have been considered stressful and have not been differentiated from “stress” associated with life-threatening events (Landys et al., 2006). The “concept of allostasis,” maintaining stability through change, allows us to distinguish between these two types of stress (McEwen and Wingfield, 2003). Daily and seasonal adjustments that maintain homeostasis within narrow life-sustaining ranges represent “allostatic state.” If environmental changes or changes in life history make the animal work harder to maintain homeostasis, an additional cost (allostatic load) is incurred. This increase in workload can be measured with overall energy expenditure. The animal enters life-threatening “allostatic overload” when energy expenditure exceeds energy intake (McEwen and Wingfield, 2003).

The combined measurement of RMSSD as a parameter for stress, and HR as an indicator for energy expenditure allowed us to assess allostatic load in our study. The decrease of HRV and HR during the winter indicate the adjustment of homeostatic set points and “allostatic state.” The decrease of HRV during spring with the associated increase in energy expenditure indicate that horses engendered an “allostatic load.”

A major limitation of our study was the small number of study-animals and the relatively high inter-individual variation in HRV. We dealt with this issue by implementing a longitudinal design that allows heterogeneity between individuals. Addressing effects of behavior and social interactions on HRV in free ranging Przewalski's horses would be of interest for future studies. Applying HRV in several wildlife species would allow for investigation of interspecific variations.

Here, we demonstrate that measuring telemetric HRV is a proven method to study undisturbed reactions of wild animals to their changing environment over the long term. Seasonal “stress” can be identified by using HRV measures, such as RMSSD, that indicate parasympathetic nervous system activity. We recommend to measure HR on a beat-to-beat basis, when collecting physiological data in future studies, in order to allow the additional assessment of HRV. We recorded cardiac activity in 3-min sequences with a self-constructed telemetric system. For future studies, the length of sequences should be increased to at least 5 min and more than one parameter for HRV should be calculated (Malik et al., 1996) i.e., estimates of overall HRV (such as SDNN, standard deviation of NN interval), long term components of HRV (e.g., SDANN), and short term components of HRV (RMSSD). Simultaneous evaluation of fecal corticoid metabolites would be of interest for method validation and comparison.

In conclusion, measuring HRV represents a promising tool to understand how animals integrate their life cycles in an ever-changing environment and potentially identify anthropogenic influences that cause stress. The combined measurement of HRV as an indicator for autonomic nervous system function and HR as an indicator for energy expenditure enables the valuation of allostatic load and “stress” in individual free-ranging mammals over a prolonged period of time.

## Author contributions

Conceived and designed the experiments: KB, CW. Designed and build transmitter: GF. Performed the experiments: KB, GS, TP, CW. Analyzed the data: FP. Wrote paper: FP and CW. Edited manuscript: KB, GF, GS, TP.

### Conflict of interest statement

The authors declare that the research was conducted in the absence of any commercial or financial relationships that could be construed as a potential conflict of interest.
