# Supplementary material for: Seasonal Variations in Heart Rate Variability as an Indicator of Stress in Free-Ranging Pregnant Przewalski's Horses (E. ferus przewalskii) within the Hortobágy National Park in Hungary
Source: Front Physiol. 2017 Sep 7;8:664. doi: 10.3389/fphys.2017.00664 (PMC5594093; doi:10.3389/fphys.2017.00664)
Supplement: Supplementary file 1 [file Table1.docx]

Supplementary Material

Seasonal variations in heart rate variability as an indicator of stress in free-ranging pregnant Przewalski’s horses (*E. ferus przewalskii*) within the Hortobágy National Park in Hungary

**Friederike Pohlin^*^, Kristin Brabender, Gerhard Fluch, Gabrielle Stalder, Thierry Petit, Chris Walzer**

*** Correspondence:** Corresponding Author: [Friederike.pohlin@gmail.com](mailto:Friederike.pohlin@gmail.com)

1. Supplementary Tables

**Supplementary Table 1.** The table provides fixed effects and a model comparison for our nested models. Model (1) includes only intercepts for each horse and a fixed effect for heart rate and months. We then stepwise add fixed effects for physiological variables: model (2) activity and body temperature; model (3) climate variables (rain, wind, humidity and outdoor temperature); and model (4) a quadratic effect for outdoor temperature (model 4). Model (4) shows the lowest AIC and hence, the best fit.

RMSSD correlates negatively with HR and body temperature, and positively with activity, rain and humidity. Outdoor temperature has a quadratic effect on RMSSD. The effect of wind is not significant. When taken all effects into account, RMSSD is still significantly lower in April than during the rest of the year. From June-October RMSSD is significantly higher than during January and in the winter. Highest RMSSD values are reached in August.

|  | | | | |
| --- | --- | --- | --- | --- |
|  | *Dependent variable:* | | | |
|  |  | | | |
|  | RMSSD.log | | | |
|  | (1) | (2) | (3) | (4) |
|  | | | | |
| Heart rate | -0.116^***^ | -0.157^***^ | -0.157^***^ | -0.157^***^ |
|  | (0.007) | (0.007) | (0.007) | (0.007) |
|  |  |  |  |  |
| Activity |  | 0.344^***^ | 0.346^***^ | 0.350^***^ |
|  |  | (0.015) | (0.015) | (0.015) |
|  |  |  |  |  |
| Body Temperature |  | -0.082^***^ | -0.045^***^ | -0.042^***^ |
|  |  | (0.008) | (0.008) | (0.008) |
|  |  |  |  |  |
| Rain |  |  | 0.159^***^ | 0.146^***^ |
|  |  |  | (0.026) | (0.026) |
|  |  |  |  |  |
| Wind |  |  | 0.008 | 0.015 |
|  |  |  | (0.011) | (0.011) |
|  |  |  |  |  |
| Outdoor Temperature |  |  | -0.083^***^ | -0.115^***^ |
|  |  |  | (0.012) | (0.012) |
|  |  |  |  |  |
| Outdoor Temperature^2^ |  |  |  | 0.061^***^ |
|  |  |  |  | (0.006) |
|  |  |  |  |  |
| Humidity |  |  | 0.009 | 0.034^***^ |
|  |  |  | (0.008) | (0.009) |
|  |  |  |  |  |
| Month (Feb) | 0.036^*^ | 0.050^**^ | 0.075^***^ | 0.142^***^ |
|  | (0.021) | (0.021) | (0.021) | (0.022) |
|  |  |  |  |  |
| Month (Mar) | -0.261^***^ | -0.239^***^ | -0.165^***^ | -0.031 |
|  | (0.021) | (0.021) | (0.023) | (0.026) |
|  |  |  |  |  |
| Month (Apr) | -1.217^***^ | -1.057^***^ | -0.881^***^ | -0.748^***^ |
|  | (0.025) | (0.027) | (0.036) | (0.038) |
|  |  |  |  |  |
| Month (May) | -0.403^***^ | -0.274^***^ | -0.091^***^ | 0.009 |
|  | (0.023) | (0.024) | (0.035) | (0.036) |
|  |  |  |  |  |
| Month (Jun) | -0.119^***^ | -0.038 | 0.164^***^ | 0.227^***^ |
|  | (0.024) | (0.024) | (0.036) | (0.037) |
|  |  |  |  |  |
| Month (Jul) | -0.040 | 0.057^**^ | 0.291^***^ | 0.297^***^ |
|  | (0.025) | (0.026) | (0.040) | (0.040) |
|  |  |  |  |  |
| Month (Aug) | 0.509^***^ | 0.610^***^ | 0.839^***^ | 0.851^***^ |
|  | (0.045) | (0.045) | (0.055) | (0.055) |
|  |  |  |  |  |
| Month (Sep) | -0.020 | 0.063^**^ | 0.270^***^ | 0.343^***^ |
|  | (0.024) | (0.025) | (0.037) | (0.037) |
|  |  |  |  |  |
| Month (Oct) | -0.062^**^ | -0.025 | 0.090^***^ | 0.209^***^ |
|  | (0.026) | (0.026) | (0.031) | (0.033) |
|  |  |  |  |  |
| Month (Nov) | -0.253^***^ | -0.186^***^ | -0.134^***^ | -0.020 |
|  | (0.021) | (0.022) | (0.023) | (0.026) |
|  |  |  |  |  |
| Month (Dec) | -0.065^***^ | -0.018 | 0.024 | 0.123^***^ |
|  | (0.024) | (0.023) | (0.025) | (0.026) |
|  |  |  |  |  |
| Constant | 0.203 | -0.128 | -0.231 | -0.379 |
|  | (0.274) | (0.264) | (0.267) | (0.267) |
|  | | | | |
| Observations | 26,687 | 26,687 | 26,671 | 26,671 |
| Log Likelihood | -30,998.970 | -30,693.140 | -30,630.670 | -30,575.780 |
| Akaike Inf. Crit. | 62,027.940 | 61,420.280 | 61,303.350 | 61,195.570 |
| Bayesian Inf. Crit. | 62,150.820 | 61,559.540 | 61,475.370 | 61,375.780 |
|  | | | | |
| *Note:* | ^*^p<0.1; ^**^p<0.05; ^***^p<0.01 | | | |
|  |  |  |  |  |

1. Supplementary Figures


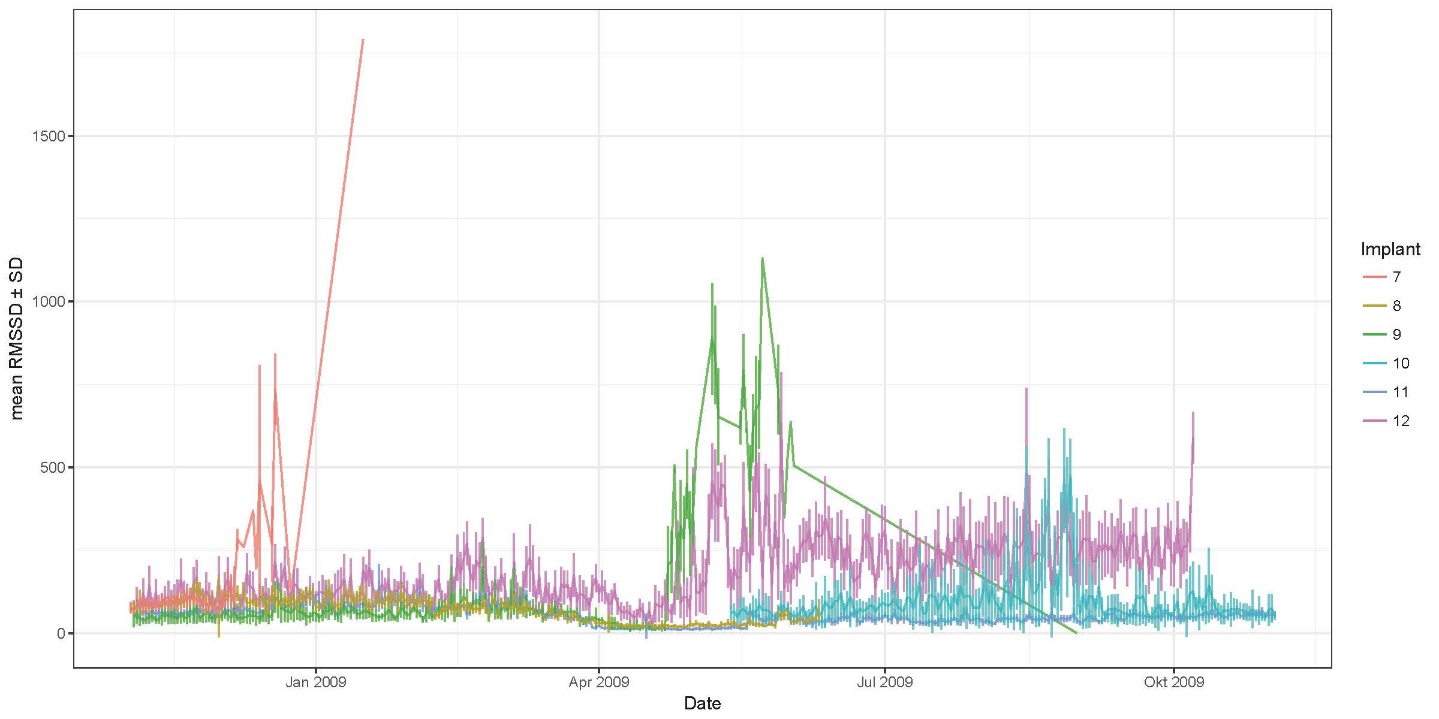


**Supplementary Figure 1.** Mean RMSSD and standard errors from 12pm-6pm from each individual throughout the 12 months study period (x-axis date (month), y-axis RMSSD (ms)). One horse lost its collar 3 months after instrumentation and two horses at approximately 10 months.


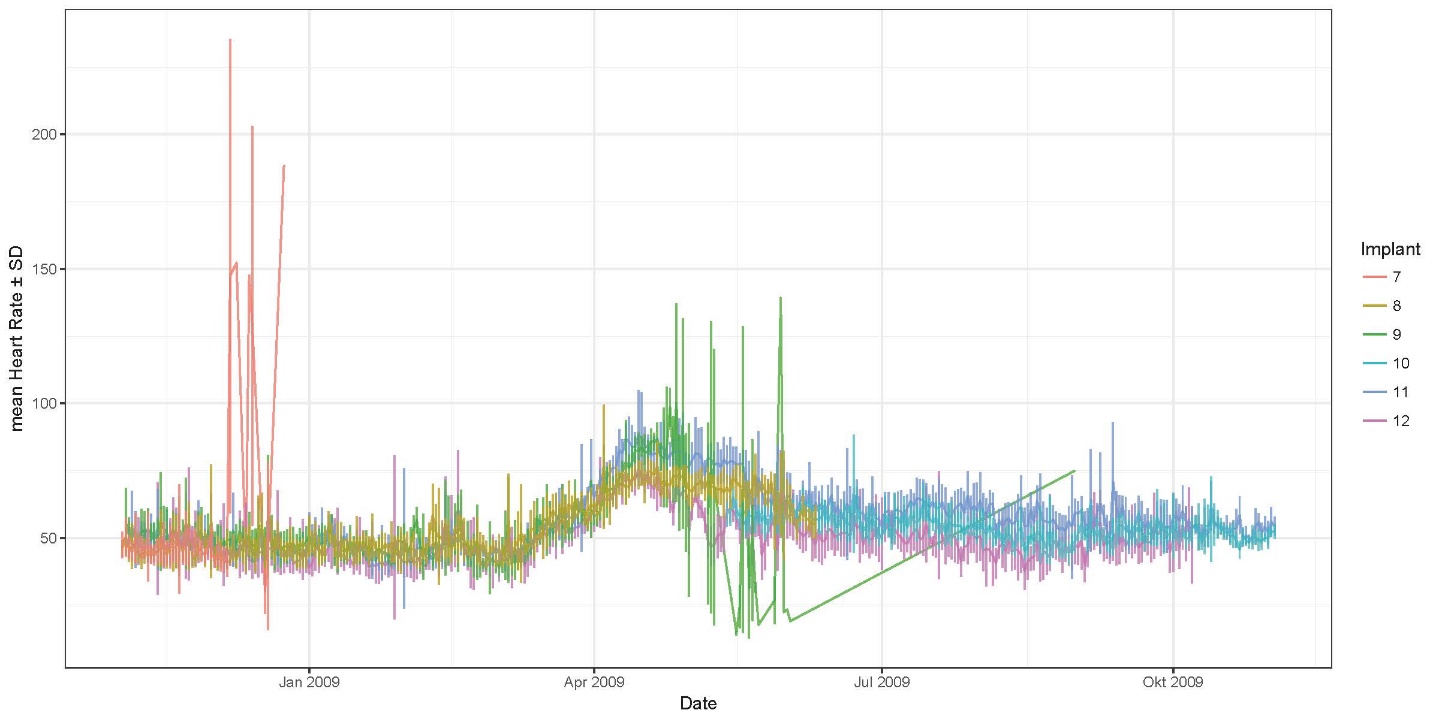


**Supplementary Figure 2.** Mean HR and standard errors from 12pm- 6pm from each individual throughout the 12 months study period (x-axis date (month), y-axis HR (bpm)). HR is lower during the winter than during the summer – it experiences a peak in spring.
